# Characterization of the decline and recovery of heat-treated *Scenedesmus vacuolatus*

**DOI:** 10.1186/1999-3110-54-3

**Published:** 2013-08-12

**Authors:** Tzan-Chain LEE, Ban-Dar HSU

**Affiliations:** Department of Life Science, National Tsing Hua University, Hsinchu, 30013 Taiwan

**Keywords:** Chloroplast, Heat stress, Programmed cell death (pcd), *Scenedesmus vacuolatus*, TEM

## Abstract

**Background:**

To find out how algal cells cope with and recover from heat stress, the small vegetative cells of the synchronous *Scenedesmus vacuolatus* culture were subjected to a heat pretreatment (46.5°C for 1 h) followed by dark recultivation. The changes in physiological activities and morphology of *Scenedesmus* cells were continuously monitored throughout the course of decline and recovery.

**Results:**

It was found that the heat treatment, though completely inhibited photosynthesis, did not kill *Scenedesmus* cells. These cells, during dark recultivation, could make a fast repair and regained the ability of proliferation. We suggest that they entered a ‘stand-by’ state, which was characterized by condensed chromatin, partially functional but morphologically altered chloroplasts, disappeared vacuoles, slightly shrunk protoplast and intact plasma membranes. These stressed cells, on the surface, seemingly were undergoing some kind of disintegration, could readily and quickly return to normal cells upon illumination. Cell death occurred only after a long period of darkness (>48 h).

**Conclusions:**

Our results suggest that the recovery of algal cells from stress damage may actually proceed in two steps. The middle “stand-by’ stage normally is gone through too rapidly to be detected unless cells are kept in the dark.

**Electronic supplementary material:**

The online version of this article (doi:10.1186/1999-3110-54-3) contains supplementary material, which is available to authorized users.

## Background

Green algae are some of the most robust organisms on earth, being able to inhabit a wide range of habitats (Tazaki et al., 1994; Lewis and Lewis, 2005). Many algae can survive harsh environmental conditions and rapidly establish new populations when conditions are favorable. For example, Desiccated desert green algae can recover high levels of photosynthetic quantum yield within 1 h of rehydration (Gray et al., 2007). Gupta and Agrawal (2006) reported that soil green alga *Rhizoclonium crassipellitum* was absent through summer to winter, and emerged in spring. Green alga, *Eremosphaera tanganyikae*, of Lake Tanganyika, East Africa, exhibited large variations in cell abundance and cell size in response to seasonal changes (Stoyneva et al., 2007). It has been shown that algae actually can reversibly enter different physiological states to accommodate the changed environment (Ferroni et al., 2009). If the environment becomes too harsh, algal cells may down-regulate their growth and repair cellular damages (Park et al., 2006; Rioboo et al., 2009), growth resumes when the environmental conditions become favorable.

High temperature is a common stress condition in nature. Heat stress can disrupt cellular homeostasis. It can also affect physiological processes such as photosynthetic activities (Allakhverdiev et al., 2008) and metabolism of reactive oxygen species (ROS; Temple et al., 2005). Generally, low doses of ROS are served as signals in cells that can activate stress protection mechanisms (Suzuki and Mittler, 2006; Møller and Sweetlove, 2010). Too large amounts of ROS however can damage cells by attacking DNA and membrane system.

There have been numerous studies on the effects of various stress on algal cells, and many of them found morphological and biochemical changes that are apoptosis-like (e.g. Moharikar et al., 2006; Zuppini et al., 2007; Segovia and Berges, 2009). However, there are many instances in nature that algal cells do not reach the point of no return in programmed cell death after experiencing stress. They may decline to a certain extent, and then followed by a full recovery. Thorough studies on this kind of process are very limited. In this study, the small vegetative cells of the synchronous *Scenedesmus vacuolatus* culture were first subjected to a heat treatment and then recultured in the dark. The physiological and morphological changes were followed in detail throughout the whole period of decline and recovery. Recultivation was done in the dark because photosynthesis was inhibited in these algae during heat pretreatment (see Results). Exposure to light even at a low intensity under this condition would lead to an overexcitation of the photosynthetic apparatus, which in turn causes photooxidative damage (Havaux et al., 1991; Öquist and Huner, 2003). Illumination during recultivation thus would act as a second stress and complicate the situation.

## Methods

### Cell culture and heat treatment

Unicellular chlorophyte *Scenedesmus vacuolatus* SAG 211-8b was cultured photoautotrophically in semi-continuous mode in a medium similar to that of Chen and Lorenzen (1986). Axenic cultures (100 mL) were grown at 32°C with slow bubbling at 3.5% CO_2_ in air. Illumination was provided by daylight fluorescence tubes at an intensity of 150 μmol photon m^-2^ s^-1^. Synchronous cultures were obtained by the programmed 14-h light/10-h dark regime. The culture started with a cell density of 2 × 10^6^ cells mL^-1^, and dilution (~15-16×) was made after each 24-h cycle, at which time cell density reached about 3 × 10^7^ cells mL^-1^. The cell number was determined using a hemacytometer under a light microscope.

When the culture was in the small vegetative cell stage, it was divided into two aliquots. Heat treatment was applied by incubating one 50-mL aliquot of small vegetative cells at 46.5°C for 1 h in the dark. The treated cells were then recultured under the same conditions as before the heat treatment, but in the dark. Another 50-mL aliquot of cells, which served as the control, was cultured in the dark directly without heat treatment. The dark cultivation lasted up to 105 h, during which samples of heat-treated and untreated cells were respectively taken for analysis at various time points as indicated below. Each experiment consisted of three independent measurements with two replicates each.

### Cell proliferation ability

The ability of cell proliferation was accessed by colony formation assay. A fixed number of cells (~ 4000 cells) was collected by withdrawing an aliquot of cell suspension at the time points of 0, 6, 8, 12 h and thereafter, at an interval of 12 h up to 84 h of dark cultivation. Samples were streaked onto agar plates containing 1.5% agar and the culture medium. The loaded plates were kept under the conditions similar to that of suspension culture (14-h light/10-h dark regime at 150 μmol photon m^-2^ s^-1^ and 32°C), but without CO_2_ supply.

The numbers of green colonies were counted 14 days later. Each plate (8.4 cm in diameter) was first photographed under a stereomicroscope, five circular areas with a diameter of 1 cm were then randomly selected and colonies in each circle were counted on a computer screen with a magnification of 10×. The number of green colonies on a plate was calculated by multiplying the average count of the five circles by the area ratio (70.56).

### Photosynthetic activity

A pulse amplitude modulated fluorometer (PAM 2000, Walz, Germany) was used to measure the in vivo chlorophyll fluorescence of *Scenedesmus* cells. The minimal chlorophyll fluorescence (Fo) was measured on dark-adapted samples with excitation at 655 nm (0.5 μmol photon m^-2^ s^-1^) and detection longer than 700 nm. The maximal fluorescence (Fm) was determined by application of a saturating pulse (< 710 nm) at 2000 μmol photon m^-2^ s^-1^ for 0.8 s. The maximum quantum yield (Fv/Fm) was calculated using the equation Fv/Fm = (Fm – Fo)/Fm.

During the heat treatment, the activity was measured at the time points of 0, 0.5, 1, 2, 4, 6, 8, 10, 15 and 20 min. During the dark cultivation, the activities of both heat-treated and untreated cells were measured at the time points of 0, 2, 6, 12 h and thereafter, at an interval of 12 h up to 84 h of dark cultivation. In addition, light (150 μmol photon m^-2^ s^-1^) was turned on during the dark cultivation of heat-treated cells at 12, 24, 36, 48, 60 and 72 h, respectively, and the Fv/Fm ratio was closely monitored at an interval of roughly 3–6 h.

### Chlorophyll content

Heat-treated or untreated cells (2 × 10^7^) were sequentially removed from the culture after cultivation in the dark for 0, 6, 12, 24, 48, 60, 72 and 85 h, centrifuged at 1,800 × g for 5 min and resuspended in 1 mL of 100% methanol. The cell suspensions were then incubated at 60°C for 30 min in the dark, followed by cooling at 0°C for 5 min, and centrifugation at 1,800 × g for 10 min. Chlorophyll concentration of the supernatant was determined photometrically by using the following equation: Chlorophyll concentration (mg L^-1^) = 25.5 × A_650_ + 4 × A_665_, where A_650_ and A_665_ are absorbance at 650 and 665 nm, respectively (Holden, 1976).

To further check the uniformity of chlorophyll content within a cell population, flow cytometry analysis was carried out with a FACSCalibur™ flow cytometer (Becton Dickinson, California). Cell samples (2 mL) of untreated cells at the beginning of dark cultivation and heat-treated cells that had been cultivated in the dark for 0, 48 and 72 h were respectively collected, and the intrinsic fluorescence of chlorophyll a was detected with excitation at 488 nm and emission longer than 650 nm. 10^4^ particles were analyzed in each measurement.

### Succinate dehydrogenase activity (MTT assay)

The succinate dehydrogenase activity assay of mitochondria was based on Ikegawa et al. (1998) with some modifications. *Scenedesmus* cells (2 × 10^7^) were collected by centrifugation at 1,800 × g for 5 min, resuspended and incubated in 1 mL phosphate buffered saline (PBS) containing 1 mg mL^-1^ 3-[4,5-dimethylthiazol-2-yl]-2,5-diphenyl-tetrazolium bromide (MTT) in the dark at 32°C for 2 h. To quantify the amount of formazan precipitate formed in a cell population, the stained cells, after PBS washes, were resuspended and incubated in 1 mL acid-isopropanol (0.04 N HCl in isopropanol) for 5 min to dissolve formazan crystals. Samples were then measured for absorbance at 590 nm. The MTT assay was done on both heat-treated and untreated cells at the time points of 0, 12, 24, 48, 78, 96 and 105 h of dark cultivation.

### Morphological studies

TEM was conducted following the procedure described by Chen and Lai (1996). *Scenedesmus* cells (6 × 10^7^) were fixed 4 h in 2% glutaraldehyde in 66mM potassium phosphate buffer (pH=7.1) at 4°C, and then post-fixed in 2% aqueous osmium tetroxide in 66 mM potassium phosphate buffer (pH=7.1) for 4 h at 4°C. This was followed by three washes at 15 min intervals with the same buffer. Fixed cells were dehydrated in a grade acetone series, then embedded in pure Spurr resin and polymerized at 70°C for 15 h. Ultrathin gold sections were cut on a Reichert-Jung ultramicrotome, collected on formvar-coated grids, stained with saturated solution of uranyl acetate in 100% methanol, and post-stained with lead citrate. Observations were made using a Hitach-7500 transmission electron microscope (Hitachi, Japan).

### Evens blue staining

*Scenedesmus* cells were incubated with Evans blue (2.5 mg mL^-1^) in 66 mM potassium phosphate buffer (pH=7.1) for 1 h at room temperature. Unbound dye was removed by three washes with the same buffer. Cell counting was carried out using a hemacytometer under a light microscope. The counting was done on both heat-treated and untreated cells at an interval of 12 h up to 108 h of dark cultivation.

## Results

### Cell proliferation ability

**Figure 1:**
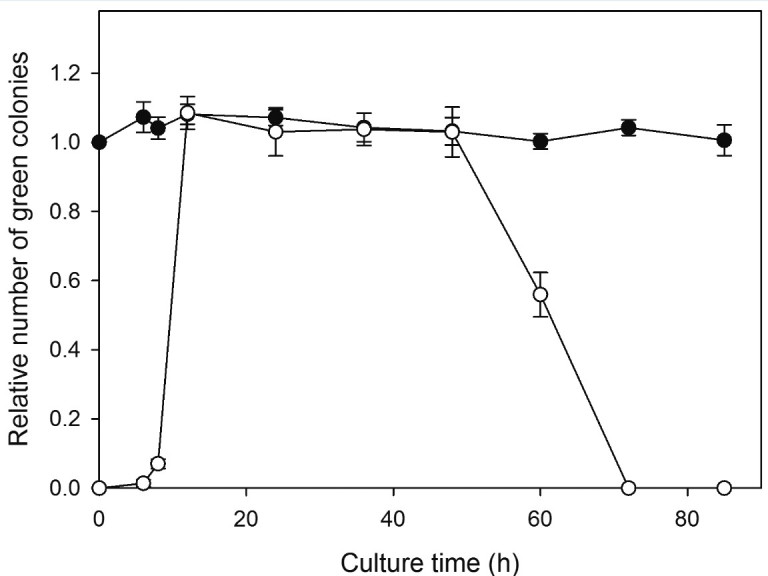
**The variation of the ability of cell proliferation.** The relative numbers of green colonies formed from seeded cells that had either been heat-treated (○) or not been treated (●) are plotted as functions of the time of dark cultivation. All the data are normalized with respect to the value of untreated cells at the beginning of dark cultivation. Each data point represents the mean ±SD of three independent measurements with two replicates each.

The numbers of green colonies formed 14 days later on agar plates were used as a measure of cell proliferation ability. Figure 1 shows the relative numbers of green colonies of heat-treated and untreated cells as functions of the time of dark cultivation. All the data have been normalized with respect to the value of untreated cells at the beginning of dark cultivation (3,467±42). Untreated cells remained viable after 84 h of dark cultivation, indicating normal *Scenedesmus* cells could survive darkness for a considerable period of time. On the other hand, all of the cells lost the ability to form green colonies right after heat treatment. A small number of green colonies appeared on the plates containing the heat-stressed cells that had been recultured for 6 h. The number of colonies then increased rapidly with culture time. After 12 h, the number of green colonies formed out of seeded cells became comparable with that of untreated cells, indicating that the heat pretreatment did not kill the algae, but a period of time was needed to do the repairing. However, after 48 h, viability started to decline rapidly, and no colony could be found longer than 72 h, suggesting that no cell could survive long term darkness.

It is worth to note, at 12 h, the colony counts of both heat-treated and untreated cells were slightly higher (about 8%) comparing to that of untreated cells at time zero (although p > 0.05). This might be attributed to the fact that synchronous cultures still contained a small number of autospore mother cells, which might be ruptured and released young vegetative cells during the process (each mother cell may contain up to 16 vegetative cells).

### Photosynthetic activity

**Figure 2:**
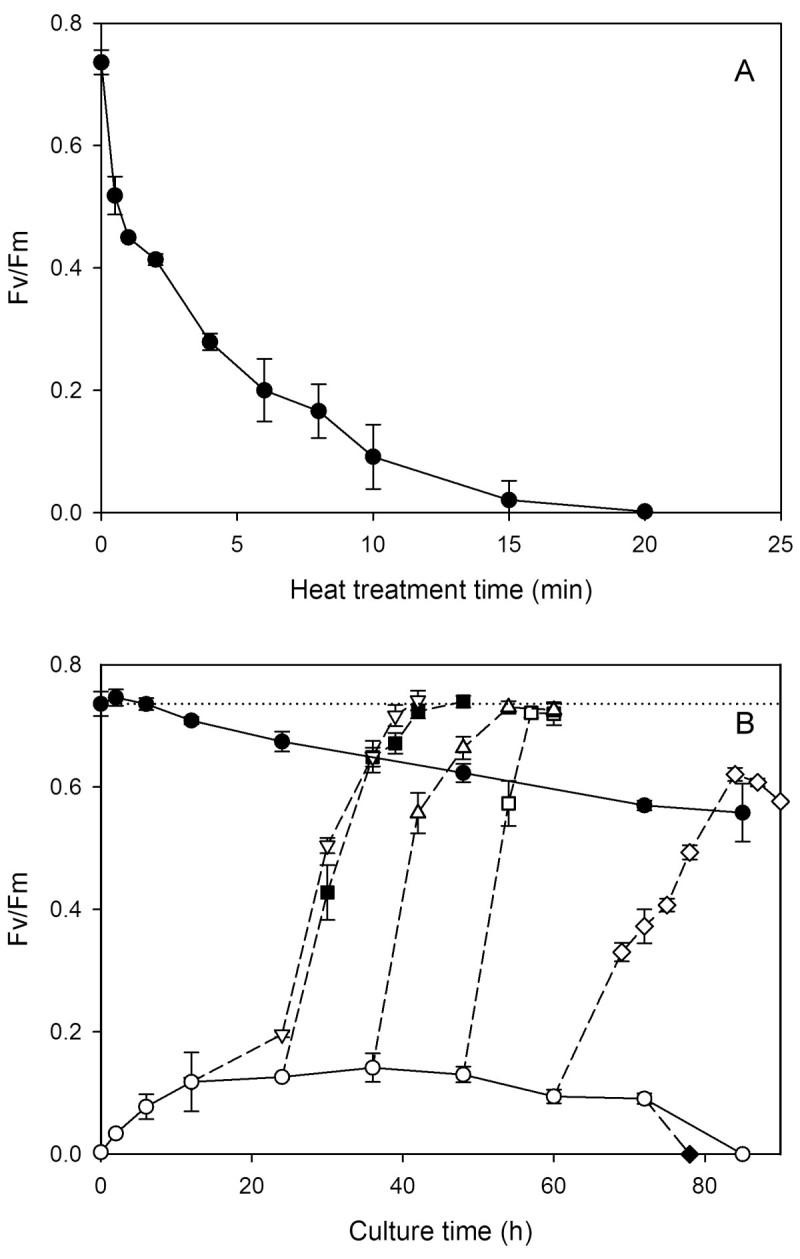
**The variation of photosynthetic activity (Fv/Fm). (A)** The decay of Fv/Fm ratio as a function of the time of heat treatment. **(B)** The variations of the Fv/Fm of heat-treated cells are plotted as functions of the time of recultivation in the dark (○), and those with light (150 μmol photon m^-2^ s^-1^) turned on at 12 (∇), 24 (■), 36 (Δ), 48 (□), 60 (◊) and 72 h (♦), respectively. The Fv/Fm of untreated cells as a function of the time of dark cultivation is also shown (●). Each data point represents the mean ±SD of three independent measurements with two replicates each. The dotted line indicates the Fv/Fm (0.74 ± 0.02) of untreated cells at the beginning of dark cultivation.

The photosynthetic activity was accessed by measuring the maximal quantum yield (Fv/Fm) of photosystem II of dark-adapted cells. The activity was completely inhibited after 20 min into the heat treatment (Figure 2A). However, as shown in Figure 2B, a quick recovery of the activity to a low level (~ 0.12) could be detected after about 12 h of dark recultivation. It then stayed steady until 48 h, but eventually declined to zero after 84 h. If light irradiation was provided sometime after 12 h, the Fv/Fm value would quickly rise to a value similar to that of untreated cells at time zero (~ 0.74), and the longer the dark period the faster the recovery. The result suggests that the repair started immediately after heat pretreatment, but a full recovery of photosynthetic activity required light. However, after 60 h of dark cultivation, the recovery upon illumination became slower and incomplete, and after 72 h, illumination simply abolished the remaining activity. The Fv/Fm of untreated cells (the control) decayed slowly during dark cultivation, and dropped only about 25% after 84 h.

**Figure 3:**
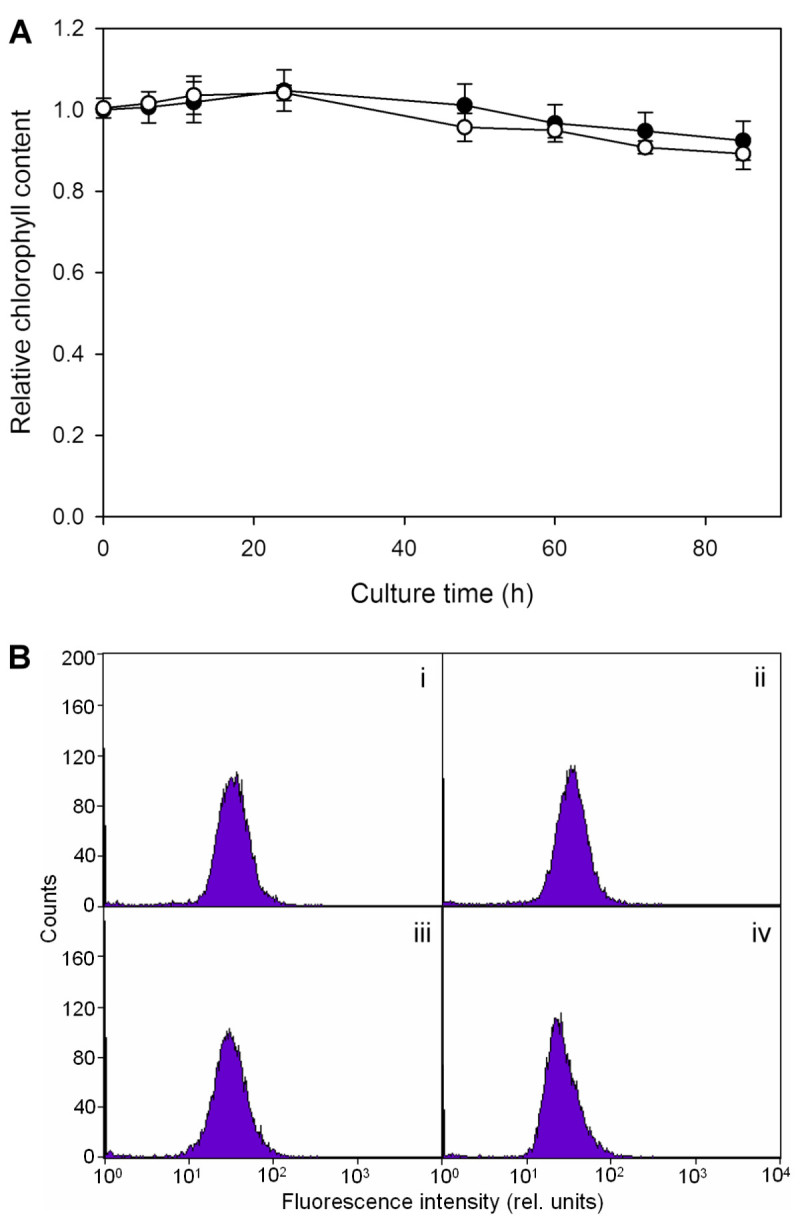
**The variation of chlorophyll content. (A)** The relative chlorophyll contents of heat-treated (○) and untreated (●) cells are plotted as functions of the time of dark cultivation. All the data are normalized with respect to that of untreated cells at the beginning of dark cultivation. Each data point represents the mean ±SD of three independent measurements with two replicates each. **(B)** The frequency distribution histograms of *Scenedesmus* cells analyzed for chlorophyll *a* fluorescence from (i) untreated cells at the beginning of dark cultivation and cells subjected to a heat treatment followed by cultivation in the dark for (ii) 0, (iii) 48 and (iv) 72 h, respectively.

As for the chlorophyll content (Figure 3A), there was no change right after heat treatment. In both heat-treated and untreated cells, slight decreases started after 24 h of dark cultivation. It dropped only about 10% after 84 h, indicating the absence of any massive chlorophyll degradation. The measurement employing flow cytometry (Figure 3B) generally confirmed the result based on methanol extraction. At 72 h (Figure 3B, iv), a small downward shift of the distribution peak position but without any change in shape indicates that the decrease in chlorophyll content was uniform across the cell population.

### Mitochondrial activity

**Figure 4:**
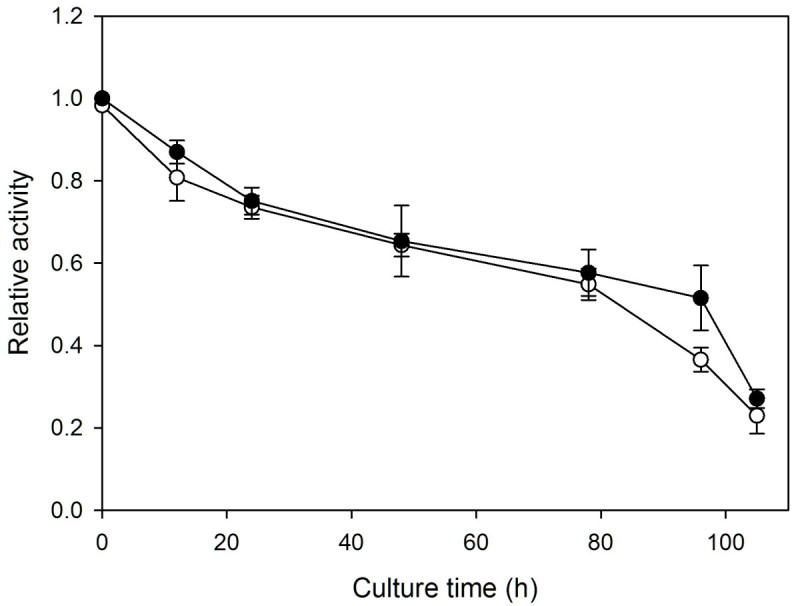
**The MTT assays for the succinate dehydrogenase activity of** ***Scenedesmus*** **mitochondria.** The relative activities of heat-treated (○) and untreated (●) cell cultures are plotted as functions of the time of dark cultivation. All the data are normalized with respect to that of untreated cells at the beginning of dark cultivation. Each data point represents the mean ±SD of three independent measurements with two replicates each.

The mitochondrial activity was accessed through one of its enzyme complex succinate dehydrogenase. As shown in Figure 4, there is no difference between treated and untreated cells at time zero, indicating that, unlike chloroplasts, the activity of succinate dehydrogenase was not affected by the heat treatment. The decline during dark cultivation was also similar between heat-treated and untreated cells. They were slow and steady at the beginning, but accelerated after 72 h. It thus seems that the loss of mitochondrial activity could be attributed solely to the darkness rather than heat treatment.

### Cell ultrastructure

#### Whole cell

**Figure 5:**
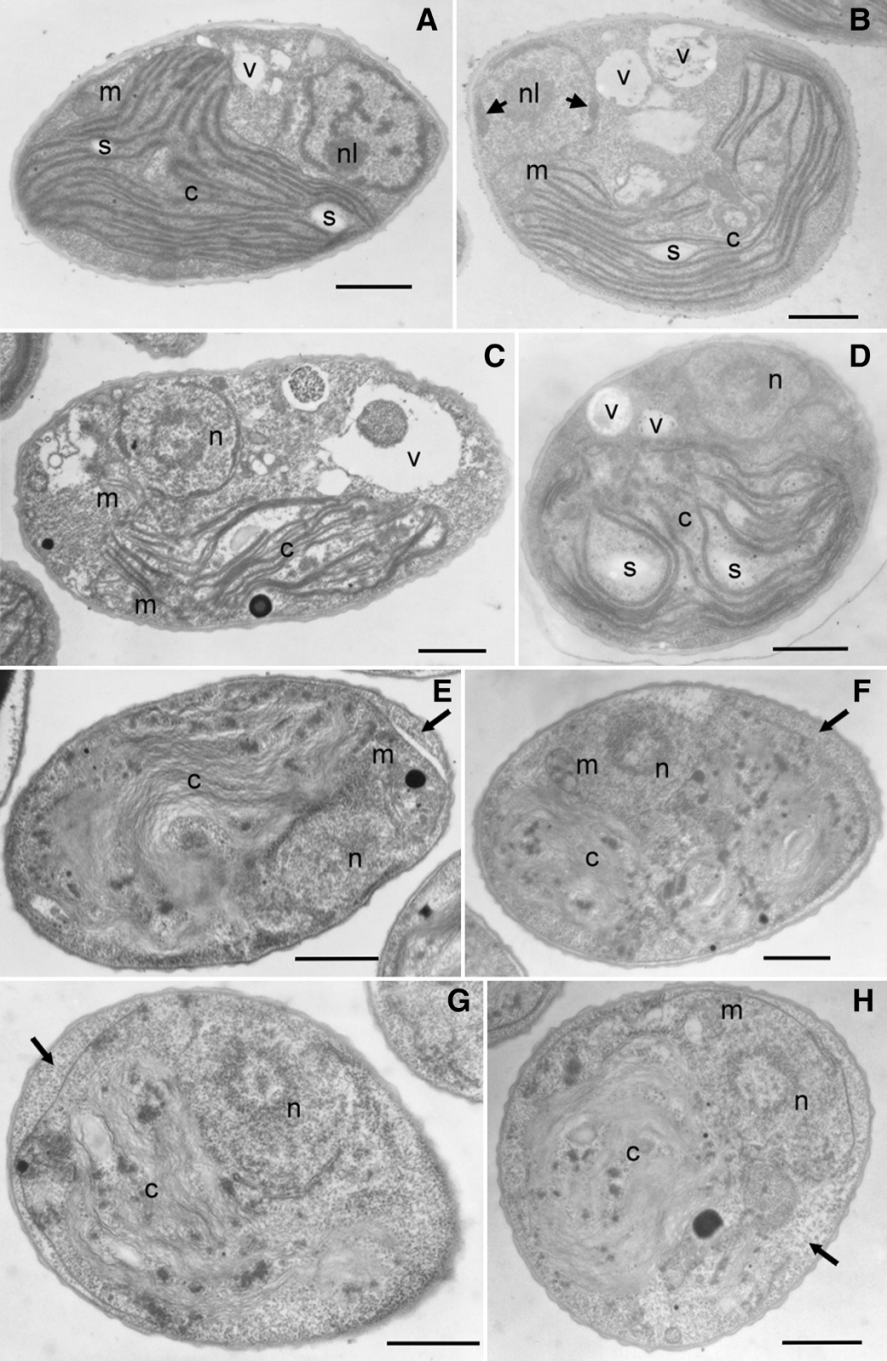
**Representative transmission electron micrographs. (A)** Untreated cell before dark cultivation (control) and the cells that had been heat treated and then cultured in the dark for **(B)** 0, **(C)** 2, **(D)** 6, **(E)** 12, **(F)** 24, **(G)** 48 and **(H)** 72 h, respectively. Bars = 1 μm. Abbreviations: c, chloroplast; m, mitochondrion; n, nucleus; nl, nucleolus; s, starch granule; v, vacuole. Arrows in **(B)**: condensation of chromatin on the inner side of the nuclear membrane. Arrows in **(E-G)**: exudation of organic substances between the cell wall and the cell membrane. Arrow in **(H)**: rupture of cell membrane.

The morphological variations were examined by TEM, and the untreated cells before dark cultivation were served as the control (Figure 5A). For heat-treated cells, there was no noticeable change in cell size, but slight shrinkage of the cytoplasm with some organic substances exuded into the space between the cell wall and the cell membrane was observed after 12 h dark cultivation (Figures 5E-G; arrows). Interestingly, the vacuole also disappeared after 12 h. The cell membranes remained intact until about 72 h, when the vast majority of cells appeared to be broken (Figure 5H, arrow).

A vital staining method with Evans blue was also used for checking the integrity of cell membranes. The staining rate at 48 h was similar to that of the control (~ 0.02%), but increased dramatically at 72 h (~ 14%) and further at 96 h (~ 21%). Although the trend of variation is similar to that observed by TEM, the values were much lower.

#### Nucleus

In the control cell, as shown in Figure 5A, the nucleolus was located in the nucleus and chromatin was dispersed in the domain surrounded by nuclear membranes. Right after heat treatment, condensation of chromatin could be seen on the inner side of the nuclear membrane (Figure 5B; arrows). The nucleolus disappeared thereafter (Figure 5C), and a ring-like structure with a diameter of about 1 μm emerged and persisted for a long time (Figures 5C-H).

#### Chloroplast and mitochondria

*Scenedesmus* cell has a cup-shaped chloroplast containing typical triplet parallel thylakoid membranes and small starch granules (Figure 5A). There was no change right after heat pretreatment (Figure 5A vs. 5B). Swelling of chloroplast was detected after dark cultivation for 6 h (Figure 5D).

**Figure 6:**
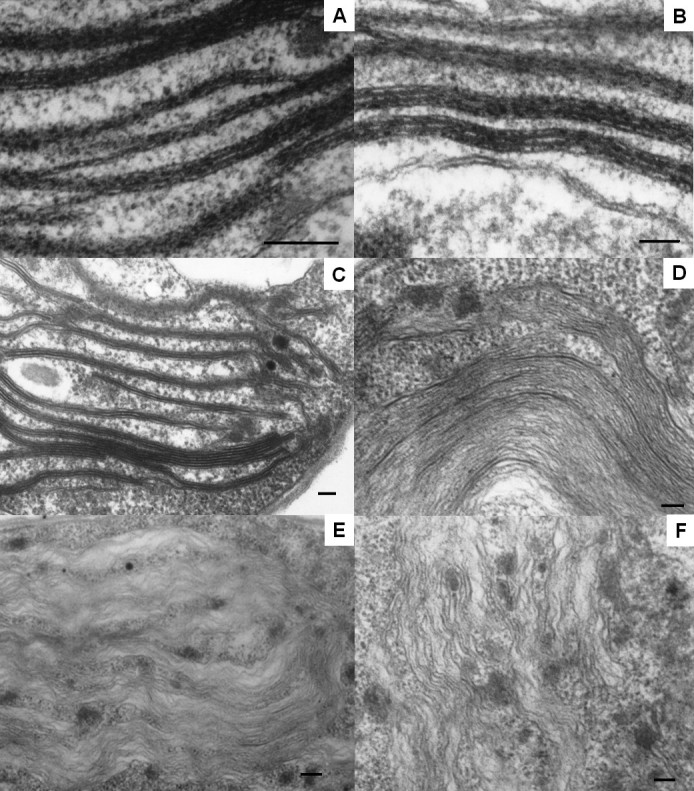
**The transmission electron micrographs of thylakoid. (A)** Untreated cell before dark cultivation (control) and the cells that had been heat treated and then cultured in the dark for **(B)** 0, **(C)** 2, **(D)** 12 h, **(E)** 24 h and **(F)** 48 h, respectively. Bars = 100 nm.

Figure 6 shows the morphological variation of thylakoid membranes in detail. They appeared less organized after 2 h (Figure 6C), and became parallel thin lines after 12 h (Figure 6D). The thylakoid membranes then lost their parallel pattern and the contrast with the background, becoming wave-like at much later hours (Figure 6E and 6F).

There was no noticeable morphological change in mitochondria after heat stress and during subsequent dark cultivation.

## Discussion

The heat pretreatment completely inhibited the light reaction of photosynthesis (Figure 2A). This is conceivable because photosynthetic activities are generally considered among the most heat-sensitive cell functions (Berry and Björkman, 1980; Yordanov et al., 1986), and photosystem II has been located as one of the primary targets of thermal damage (Allakhverdiev et al., 2008).

During the subsequent dark cultivation, there were several significant changes occurring in these heat-stressed cells, but the majority of them remained alive and, after a short delay, regained and retained the proliferation ability until after 48 h (Figure 1). Margination and condensation of chromatin was detected at the early stage of dark cultivation (0–2 h, Figure 5B and 5C). Similar phenomena were observed in many other stressed unicellular algae, and often regarded as one of characteristic features of programmed cell death (Segovia et al., 2003; Zuppini et al., 2007). This was certainly not true in our case. Chromatin condensation, a packing state which is normally associated with loss in transcription capacity, actually may provide protection from environmental stress.

Chloroplasts underwent dramatic alternations during dark cultivation. In the absence of noticeable chlorophyll degradation (Figure 3), photosynthetic activity recovered to a low level in 12 h (Figure 2B), which coincided with the full regaining of cell proliferation ability (Figure 1), and thereafter could quickly return to the same value as the control upon illumination. This recovery ability lasted until 48 h. It seems that the repair of photosynthetic apparatus started along with cultivation, and comprised two stages. The first could be done in the dark, and the second required light irradiation (van Wijk et al., 1994; Minagawa and Takahashi, 2004). Morphologically, the most noticeable change also occurred around 12 h, when the thylakoid membranes transformed from dense and thick into parallel thin lines (Figures 5E and 6D), which was most likely to be due to decomposition of membrane proteins. It looks on the surface that the thylakoids were on the way to disintegration (e.g. Kratsch and Wise, 2000; Gabara et al., 2003; Zuppini et al., 2007). Nevertheless, many features that characterize senescent chloroplasts such as decrease in volume, disappearance of thylakoid membrane system, increases in the size and number of plastogluobuli and degradation of chlorophyll were absent (Krupinska, 2007). These chloroplasts were actually able to exhibit a low level of activity and make a full recovery upon illumination. We suggest that the chloroplasts, in between 12 and 48 h of dark cultivation, entered a ‘stand-by’ state, in which the cell maintained intact chloroplasts and functional photosynthetic machinery, so that a quick recovery can resume when light became available. Mitochondria, on the other hand, were not affected by heat treatment. A similar decay rate of both heat-treated and untreated cells also suggests that the decline of the activity was not a post effect of heat treatment, but the result of darkness (Figure 4). Taking also into account the absence of noticeable change in morphology during dark cultivation, it seems that mitochondrion did not play a determining role in cell viability.

An analogue can be seen in higher plants. Although darkness is commonly considered to be an inducer of leaf senescence, this process is actually inhibited when whole plant is placed in the darkness (Weaver and Amasio, 2001). In whole darkened plants of Arabidopsis, the photosynthetic capacity is maintained, whereas the capacity of mitochondrial respiration decreases (Keech et al., 2007). It has been suggested that the metabolism in leaves of these whole darkened plants enters a ‘stand-by mode’ to preserve the photosynthetic machinery for as long as possible. For *Scenedesmus vacuolatus*, a cell is a whole organism.

Other noticeable changes that also occurred after 12 h of dark cultivation included disappearance of vacuole and shrinkage of the protoplast (Figure 5). Rupture of the tonoplast is a common phenomenon in plant programmed cell death. It has been observed during the senescence of leaf cells, the development of phloem cells, as well as the formation of aerenchyma and root cap cells (von Doorn and Woltering, 2005). The common result of these processes is degradation of cellular constituents by the released vacuolar hydrolases. This however was not detected in heat-stressed *Scenedesmus* cells, showing a clear distinction between the two. Detachment of the cell membrane from the cell wall is frequently observed in stressed cells, especially those going through necrosis (van Doorn et al., 2011). However, unlike necrotic cells, stressed *Scenedesmus* cells maintained their cell membrane integrity until after 72 h of cultivation. It is worth to note that, after detachment, the space between the cell wall and the cell membrane was filled with organic substances. This might be the degradation products of cellular constituents, or simply some extracellular substances produced by cells because algae are known to increase the production of such organic substances under stress (Maršalek and Rojıcková, 1996; Mishra and Jha, 2009).

The ‘stand-by’ state of *Scenedesmus* cells could not last for long in darkness. After 48 h of cultivation, cells quickly lost proliferation ability (Figure 1), and the photosynthetic activity also started to decline (Figure 2). Note that after 60 h, the recovery of photosynthetic activity induced by illumination became slower and incomplete, and after 72 h, illumination only brought about inhibition. Disruption of plasma membrane also occurred after 72 h (Figure 5H). We suggest that energy consumption continued even when cells switched to the ‘stand-by’ state. Maintenance of membrane integrity expended energy. Death occurred when cell’s energy source was depleted.

## Conclusions

The heat treatment at 46.5°C would not kill *Scenedesmus* cells. In darkness, the population made a fast repair. Cells then entered a ‘stand-by’ state, which was characterized by condensed chromatin, partially functional but morphologically altered chloroplasts, disappeared vacuoles, slightly shrunk protoplast and intact plasma membranes. They could quickly return to normal cells upon illumination. Cell death occurred only after a long period of darkness (>48 h). These results seem to suggest that the recovery of algal cells from stress damage may actually proceed in two steps. The middle “stand-by’ stage normally is gone through too rapidly to be detected when cells are under illumination. It becomes observable only when recovery is allowed to proceed in the dark.

## Authors’ original submitted files for images

Below are the links to the authors’ original submitted files for images. Authors’ original file for figure 1 Authors’ original file for figure 2 Authors’ original file for figure 3 Authors’ original file for figure 4 Authors’ original file for figure 5 Authors’ original file for figure 6
