# Supplementary figures and images for: Characterization of the decline and recovery of heat-treated Scenedesmus vacuolatus
Source: Bot Stud. 2013 Aug 12;54:3. doi: 10.1186/1999-3110-54-3 (PMC5383920; doi:10.1186/1999-3110-54-3)

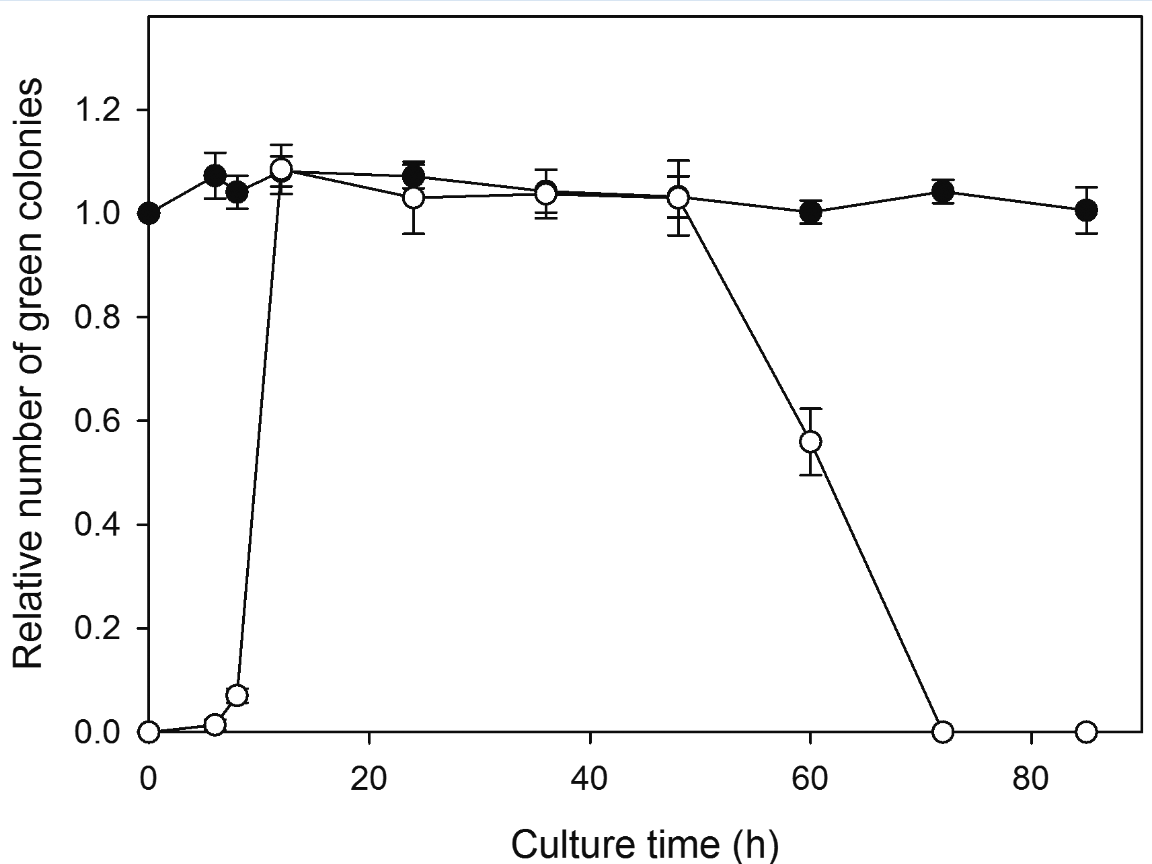

Supplement: Supplementary file 1 — Authors’ original file for figure 1 [file 40529_2012_3_MOESM1_ESM.png]

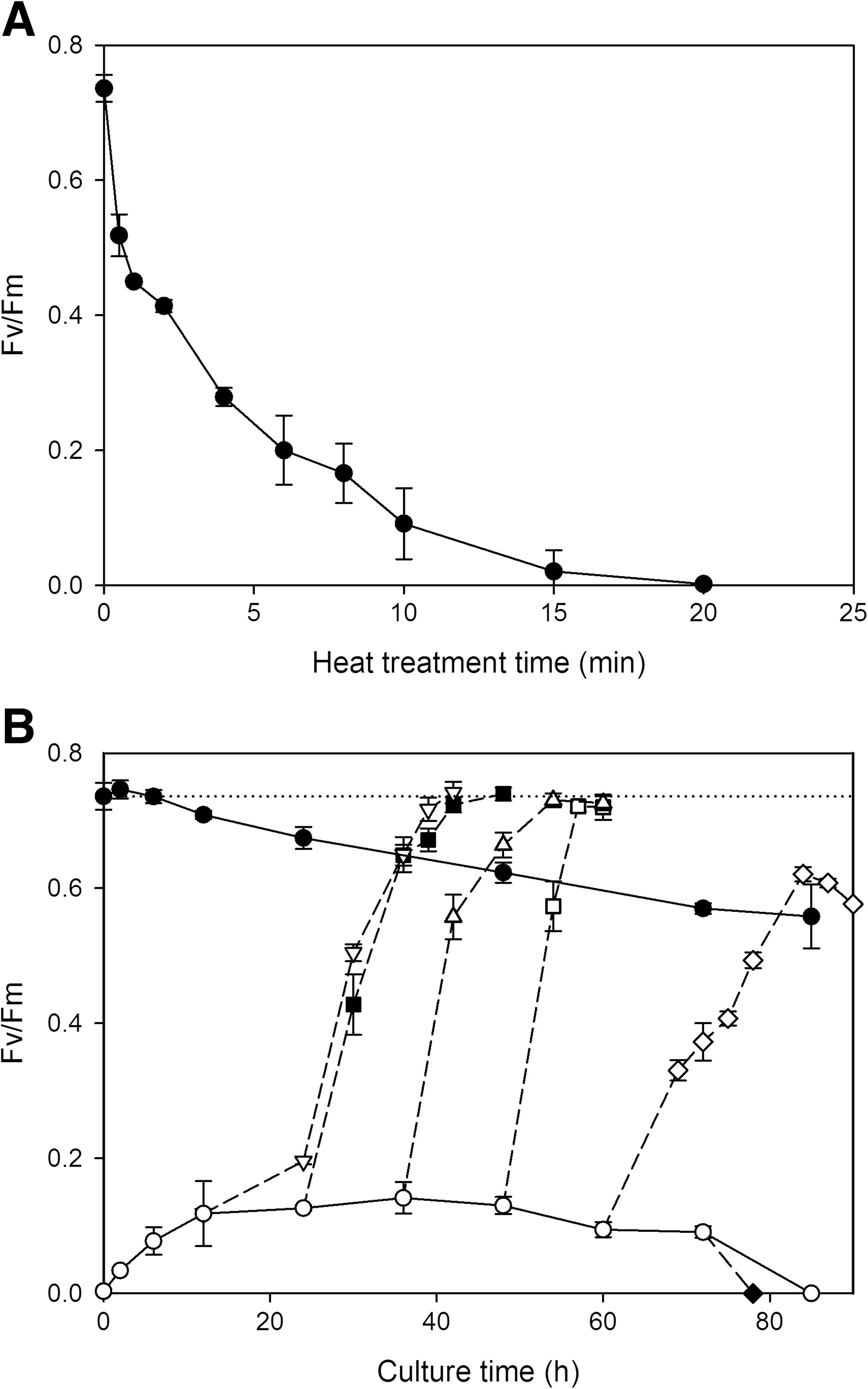

Supplement: Supplementary file 2 — Authors’ original file for figure 2 [file 40529_2012_3_MOESM2_ESM.tiff]

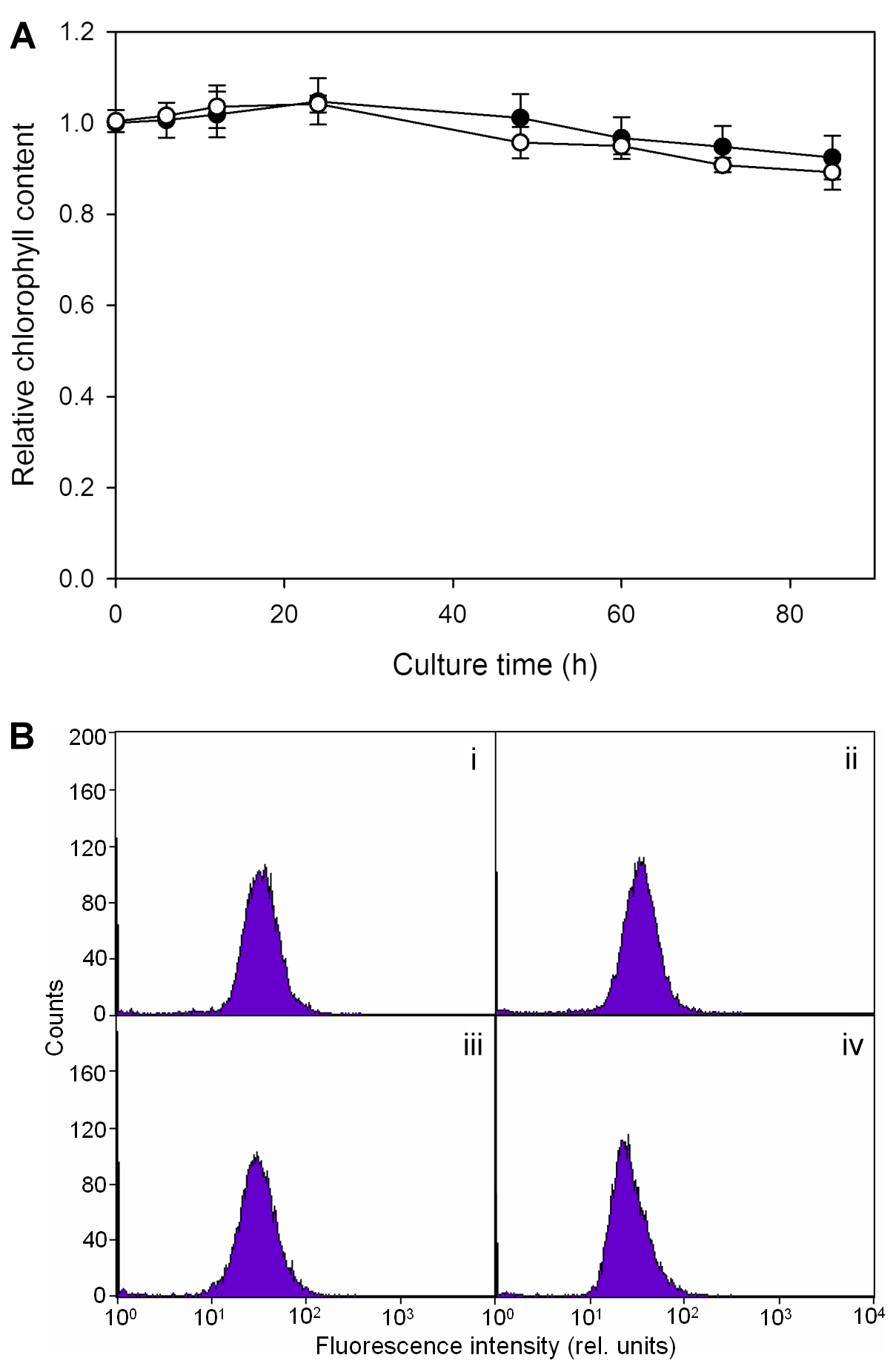

Supplement: Supplementary file 3 — Authors’ original file for figure 3 [file 40529_2012_3_MOESM3_ESM.tiff]

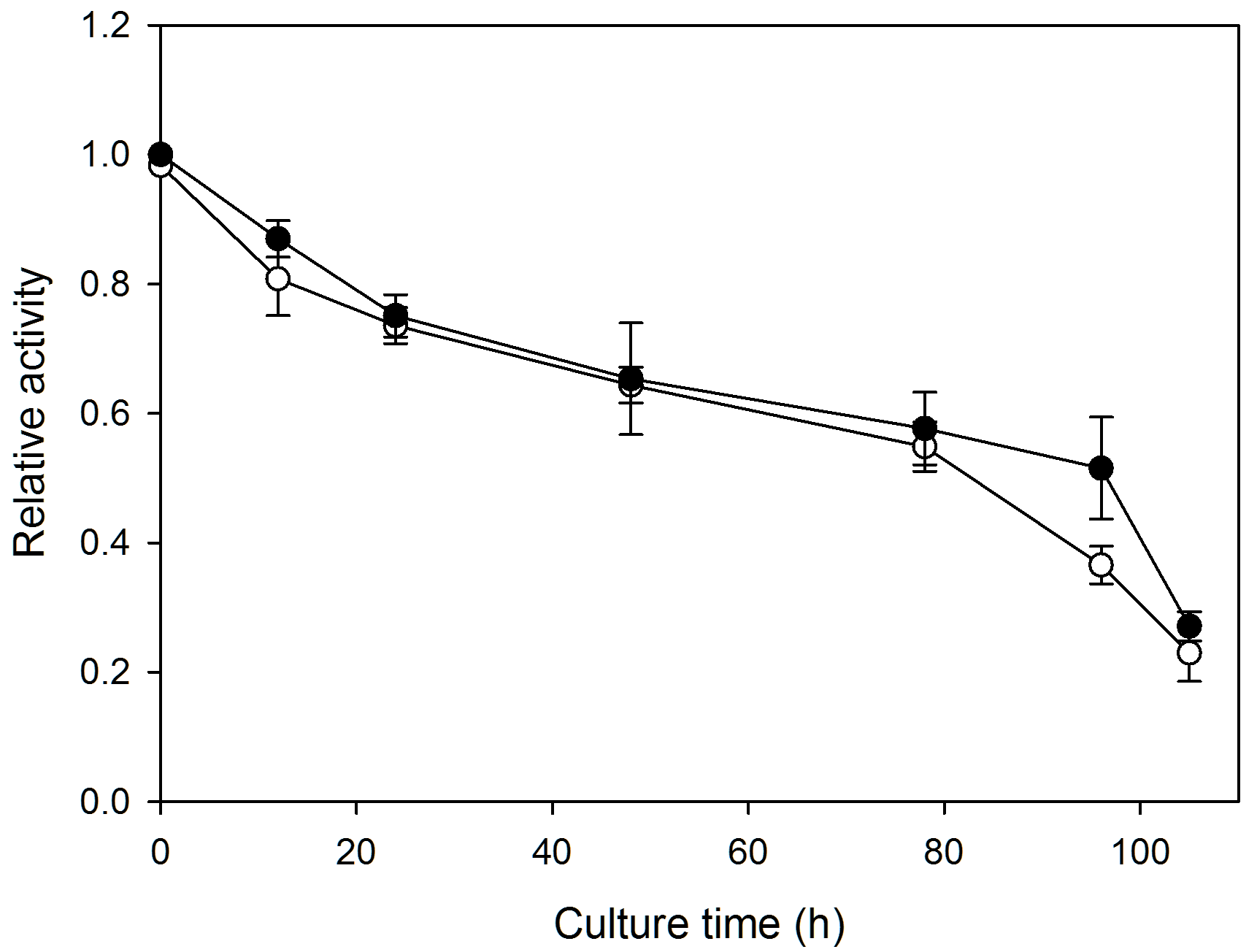

Supplement: Supplementary file 4 — Authors’ original file for figure 4 [file 40529_2012_3_MOESM4_ESM.tiff]

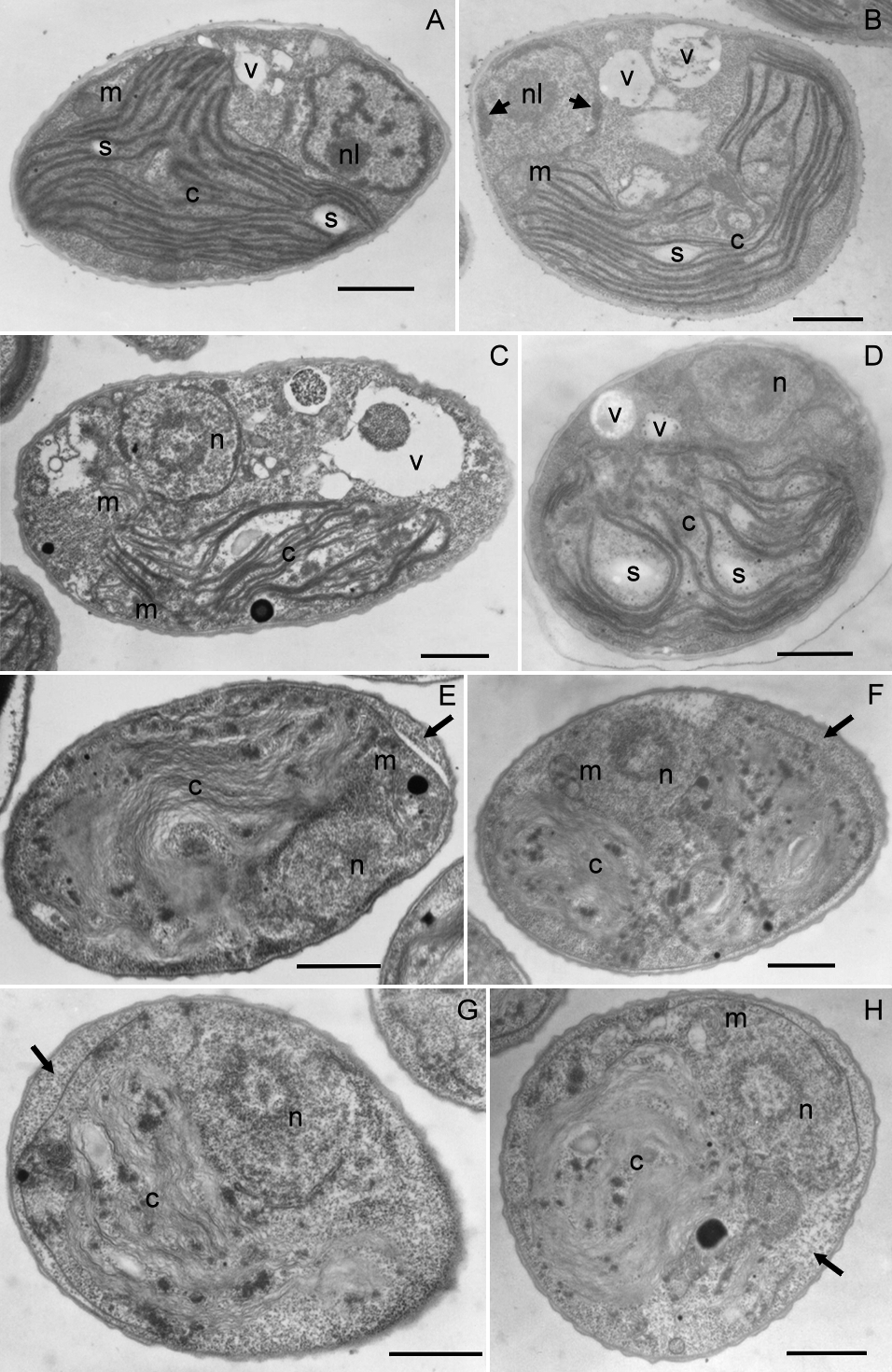

Supplement: Supplementary file 5 — Authors’ original file for figure 5 [file 40529_2012_3_MOESM5_ESM.tiff]

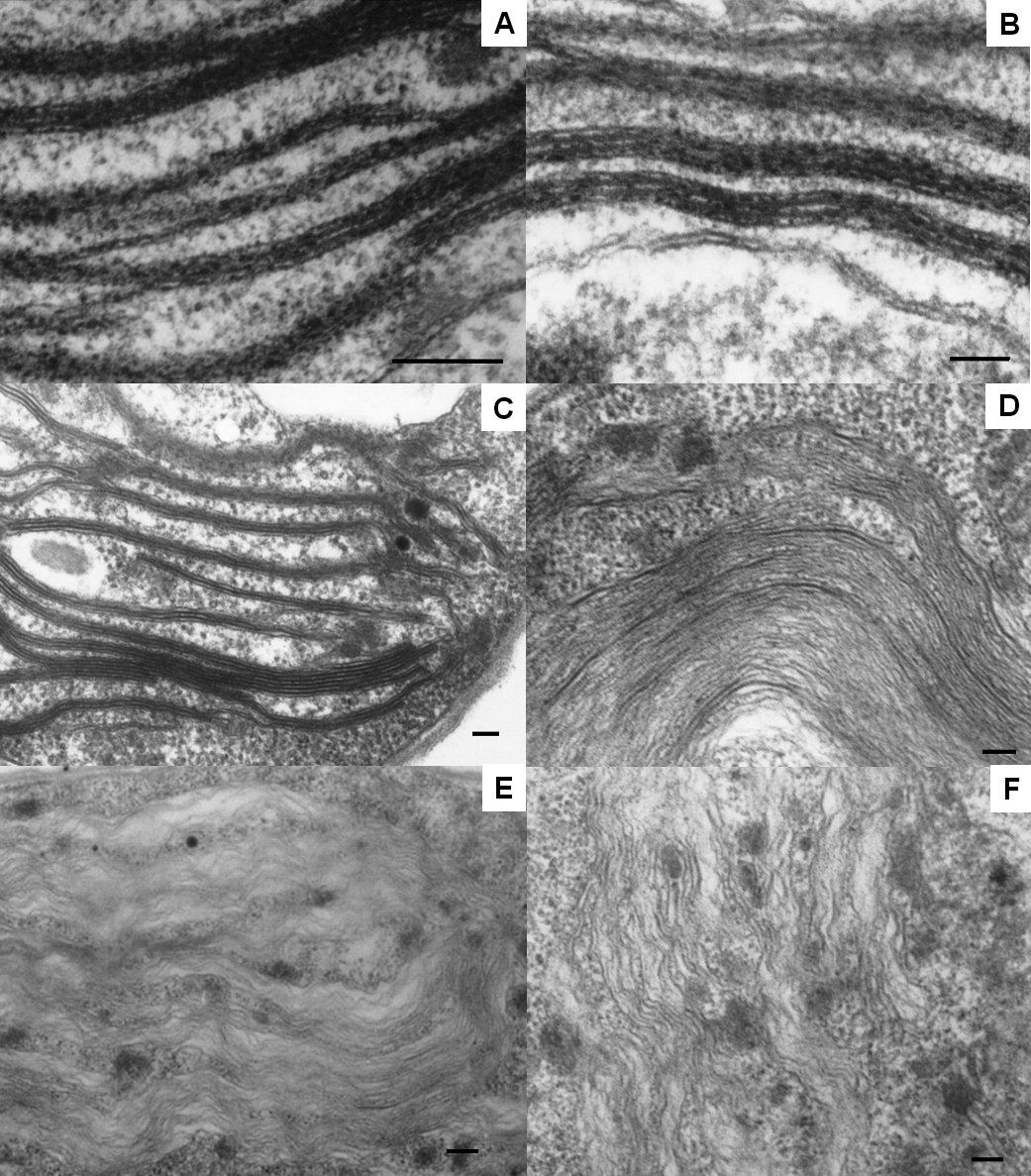

Supplement: Supplementary file 6 — Authors’ original file for figure 6 [file 40529_2012_3_MOESM6_ESM.tiff]
